# CAREGIVERS’ PERCEPTION DISCREPANCY IN THE CAREGIVING CONTEXT: THE IMPACT ON PSYCHOLOGICAL WELL-BEING

**DOI:** 10.1093/geroni/igad104.1137

**Published:** 2023-12-21

**Authors:** Yulri Kim, Jeong Eun Lee, Joseph Svec, Natasha Nemmers

**Affiliations:** Iowa State University, Ames, Iowa, United States; Iowa State University, Ames, Iowa, United States; Saint Joseph’s University, New York City, New York, United States; Wayne State University Institute of Gerontology, Detroit, Michigan, United States

## Abstract

Guided by the social exchange perspective, caregiving involves a reciprocal exchange in which caregiver perceptions influence a bevy of positive and negative outcomes. Positive perceptions correspond with enhanced psychological well-being (Ejem et al., 2018; Call et al., 1999). However, those who perform high levels of care but feel undervalued may experience poorer psychological well-being compared to those who perform lower levels of care but feel appreciated (Nah et al., 2022). The matrix of outcomes, contingent on the balance of performance and appreciation, highlights how perception discrepancies in the caregiving context can explain variations in psychological well-being among caregivers. In this study, we examine the psychological well-being of caregivers in four distinct groups based on their caregiving activities (high/low) and their perceived appreciation (high/low) of their efforts from care recipients. We analyze 2,096 informal caregivers who were included from Round 5 of the National Study of Caregiving (NSOC). Utilizing an analysis of covariance, the results indicate that high activity caregivers with low perceived appreciation had poorer psychological well-being than other groups. Additionally, caregivers with low caregiving activities and high perceived appreciation have higher psychological well-being than caregivers with high care activity and high perceived appreciation. These findings highlight the significance of perceived appreciation from care recipients for improving psychological well-being of caregivers.

